# Do household asset wealth measurements depend on who is surveyed? Asset reporting concordance within multi-adult households in rural Uganda

**DOI:** 10.7189/jogh.10.010412

**Published:** 2020-06

**Authors:** Meghan L Smith, Bernard Kakuhikire, Charles Baguma, Justin D Rasmussen, David R Bangsberg, Alexander C Tsai

**Affiliations:** 1 Boston University School of Public Health, Boston, Massachusetts, USA; 2 Mbarara University of Science and Technology, Mbarara, Uganda; 3 Massachusetts General Hospital, Boston, Massachusetts, USA; 4 Oregon Health & Science University-Portland State University School of Public Health, Portland, Oregon, USA; 5 Harvard Medical School, Boston, Massachusetts, USA

## Abstract

**Background:**

In resource-limited settings, the Filmer & Pritchett asset index is frequently used to measure household economic status. Little is known about how its validity is affected by differential reporting or recall within households.

**Methods:**

As part of a whole-population survey in a rural region of southwestern Uganda, we elicited household asset information from married dyads (404 men and 404 matched women) residing within the same households. We assessed the extent to which the asset index yielded differing measures of relative household wealth, depending on whether the husband’s or wife’s survey data were used in its calculation. To estimate agreement, we used Cohen’s κ for binary and categorical variables, and Cronbach’s α for continuous variables. We also assessed the extent to which asset wealth quintiles assigned based on husbands’ vs wives’ reporting were concordant, and whether discordance was related to demographic characteristics.

**Results:**

For most individual assets, agreement ranged from moderate to very good. Asset index scores based on husbands’ vs wives’ reporting were positively correlated (Pearson *r* = 0.85). Corresponding wealth quintiles were moderately concordant (weighted κ = 0.65); 171 households (43%) differed by one or more quintiles when the husbands’ vs wives’ reporting was used, and 43 (11%) differed by two or more quintiles. Concordance in asset wealth quintile could not be explained by joint educational attainment, age, or age difference.

**Conclusions:**

There is significant intra-household variability in household asset reporting that can materially affect how households are classified on a widely used measure of relative household asset wealth.

In settings where the local economy is dominated by animal husbandry or subsistence agriculture, traditional measures of economic status (eg, wage income or consumption expenditure) may not accurately measure livelihood status or economic well-being. The household asset index, or wealth index, proposed by Filmer & Pritchett [1,2] has emerged as a useful alternative. The asset index combines information on multiple household assets and housing characteristics into a single continuous measure, which can then be used to rank households in relative terms, typically by quintiles [1]. It is based on easily observable variables (eg, asset ownership and housing characteristics) and is therefore thought to be less subject to recall error compared with variables that have significant seasonal variation or that are more difficult to measure (eg, wage income). In resource-limited settings where the local economy is dominated by animal husbandry or subsistence agriculture, the asset index is thought to better capture inter-household variation in longer-term economic well-being compared with traditional measures [1,3,4].

The asset index has been used to measure economic status in diverse settings [5-7], and generally is considered to have good validity [1]. It has been incorporated into the Demographic and Health Surveys as a standard measure [8] and is routinely used in analyses of socioeconomic inequalities in health and health behavior [9-11]. However, little is known about intra-household variation in household asset reporting and the extent to which such variation may potentially influence measurement of household-level indicators of economic well-being. Household surveys often involve the participation of multiple members of a household, but in many of these surveys – including the Demographic and Health Surveys [8] – household asset information is elicited only from the head of the household, typically a man.

There may be sex-based discrepancies in asset reporting related to societal expectations, social desirability, differential use of assets, differential access to assets, or differential knowledge of assets. Related work has documented a lack of spousal concordance in reports about labor productivity [12], as well as lack of concordance in reporting abouta wide range of health-related matters, including reproductive health [13], fertility decisions [14], and intimate partner violence [15-18]. The studies most relevant to ours are those that examine concordance between husbands and wives in reports of land ownership [19,20] and food/water insecurity [21,22]. However, while these studies have considered variation in reporting to represent true differences in access to resources, we consider household wealth to be is a single shared construct measured with varying amounts of error depending on the respondent.

To address these gaps in the understanding of intra-household variation in asset reporting, we analyzed data from a whole-population survey conducted in a rural area of southwestern Uganda to assess the extent of agreement in asset reporting between married men and women residing within the same households. We calculated an asset index based on the asset reporting of each participant and then compared the extent to which discrepancies in husbands’ vs wives’ reporting resulted in their household being categorized into different asset wealth quintiles. Finally, we assessed agreement in household wealth status and estimated correlates of agreement based on joint demographic characteristics of the married dyads.

## METHODS

### Sample

The study was conducted in Mbarara District, a rural area of southwestern Uganda approximately 260 km southwest of Kampala. Participants were drawn from a whole-population sample of Nyakabare Parish, a rural administrative subunit located approximately 20 km outside of Mbarara District’s commercial center. Nyakabare is comprised of eight villages, and residents’ livelihoods are derived principally via subsistence farming, animal husbandry, petty trading, and supplemental migratory work. Food and water insecurity are common in this setting [22-24].

All adults aged 18 years and older, and emancipated minors aged 16 to 18 years, who were stably residing in Nyakabare were enumerated for the study. Residents were excluded if they were unable to provide informed consent or communicate meaningfully with research staff due to psychosis, acute intoxication, neurological damage, deafness, or other reasons, as determined by supervised non-clinical research staff in the field.

Research assistants fluent in Runyankole interviewed consenting participants between June 3, 2014, and August 14, 2015. Potential participants were approached to assess their interest in the study. After describing the purpose of the study, informed consent was obtained. Research assistants verbally reviewed the informed consent document with potential study participants, who were probed for comprehension and given opportunities to ask questions. Study participants who could not read and/or write were permitted to indicate consent with a thumbprint.

Of 1816 invited participants, 1779 (98%) agreed to take part in the study. These participants resided within 767 unique households. The present analysis was restricted to households in which one or more married man and woman resided (n = 487). In households with more than one married couple (n = 11), the oldest couple was selected. In polygamous households (n = 13), the oldest wife was included. Households were excluded from the analysis if either the husband or the wife was missing data on household assets (n = 59), leaving 404 households for the analysis. Thus, 404 men and 404 matched women were included in the analysis. All participants were interviewed separately, even if they resided within the same household.

### Measures

#### Household asset wealth index

Participants were asked to report whether or not their household had the following possessions: radio, lantern, bicycle, television, iron (ie, iron box), motorbike (*boda boda*), refrigerator, stove, or car. They were also asked about type of toilet facility; predominant materials used in the construction of the household floors and walls; number of land plots owned; number of rooms in the home; number of mobile phones owned; number of cows, chickens, and goats owned; and size of the household's rainwater harvesting tank, if any.

Following the methods proposed by Filmer and Pritchett [1,2] (which have been broadly disseminated throughout the Demographic and Health Surveys [8]), we applied principal component analysis to these asset variables, first using only data from the men, and then using only data from the women. The first principal component defined the household asset wealth index. Similar to previous studies [25], in this sample, the first principal component explained 22% of the variance in household asset wealth as reported by men and 24% of the variance among women. While absolute values of the asset index carry no substantive meaning, the values can be used to rank households in terms of their asset wealth relative to other households in the community. Thus, we used asset index scores to categorize households into quintiles of asset wealth (“wealth quintiles”).

#### Sociodemographic variables

In addition to sex, sociodemographic variables included highest level of education completed (none, some primary, completed primary, or more than primary) and age in years. We used this information to create variables corresponding to husband/wife dyads: completion of primary education by both husband and wife, by neither, by husband only, and by wife only; both husband and wife younger than 30 years of age, both husband and wife between 30 and 49 years of age, both husband and wife older than 49 years of age, and mixed age groups; and gap (in years) between the husband’s and wife’s ages.

### Analysis

We tabulated individual sociodemographic characteristics of participants by sex, as well as joint characteristics of the husband-wife dyads. For individual assets, we calculated percent agreement between husbands and wives, and calculated Cohen’s kappa (κ) and corresponding 95% confidence intervals (CI) to account for the possibility of agreement occurring by chance. For ordinal asset variables (ie, rainwater harvesting tank size, number of mobile phones), we calculated weighted κ, which accounts for chance agreement but also weights according to degree of disagreement on the ordinal scale. For assets measured on the continuous scale (number of land plots, rooms in the home, cows, chickens, and goats), we calculated the intraclass correlation (3,k) – equivalent to Cronbach’s alpha [26] – to assess agreement. To assess agreement in wealth quintile categorization, we used weighted κ as described above.

To estimate correlates of concordance in wealth categorization, for each husband-wife dyad we created a binary variable equal to 1 if asset reporting from both the husband and wife resulted in their household being categorized into the same wealth quintile, vs 0 otherwise (ie, asset reporting from the husband and wife resulted in the household being categorized into different wealth quintiles). Specifying this variable as the outcome in a multivariable log-binomial regression model, we estimated the extent to which dyadic demographic characteristics were correlated with concordance in wealth categorization.

Although quintiles are overwhelmingly used in the literature as a categorical measure of household asset wealth [1,8], we sought to ensure that our findings were not driven by the arbitrary selection of quintiles as the categories of interest. Therefore, we conducted a sensitivity analysis using three alternate methods of categorizing the continuous asset index scores: (1) bottom 40%, middle 20%, and top 40% (15); (2) bottom 20%, middle 60%, and top 20% [27]; and (3) bottom tertile, middle tertile, and top tertile [28].

All analyses were conducted using SAS Version 9.4 (IBM Inc, Armonk, NY, USA).

### Ethics

Ethical approval for this study was received from the Partners Human Research Committee at Massachusetts General Hospital and the Research Ethics Committee at Mbarara University of Science and Technology.

## RESULTS

### Descriptive results

Men were older than women on average (**Table 1**), but husbands’ ages generally increased linearly with the ages of their wives. The median age difference between the husband and wife in a given household was 7 years (interquartile range, 7 years). Men were more likely than women to have completed a primary education (59% vs 49%). In 144 households (36%), both the husband and wife completed primary school, but there were more households in which only the husband completed primary school (96 [24%]) than households where only the wife completed primary school (53 [13%]).

**Table 1:** Summary statistics

	Husbands (N = 404)		Wives (N = 404)		Dyads (N = 404)	
	**N**	**%**	**N**	**%**	**N**	**%**
**Age (years):**						
<30	52	13	144	36	-	-
30-39	113	28	115	28	-	-
40-49	105	26	82	20		
>49	134	33	63	16		
**Educational attainment:**						
Completed primary education or more	240	59	197	49	-	-
Completed less than primary education	164	41	207	51		
**Joint age (years):**						
Both <30	-	-	-	-	50	12
Both 30-49	-	-	-	-	130	32
Both >49	-	-	-	-	63	16
Mixed ages	-	-	-	-	161	40
**Joint education:**						
Both completed primary education or more	-	-	-	-	144	36
Only husband completed primary education or more	-	-	-	-	96	24
Only wife completed primary education or more	-	-	-	-	53	13
Neither completed primary education	-	-	-	-	111	27

### Agreement for individual assets

For the 12 assets coded as binary variables, percent agreement (ie, both husband and wife endorsed, or both did not endorse, an item as being present in the household) ranged from 82% for lantern to 98% for refrigerator and type of toilet facility (**Table 2**). There were few substantive differences in endorsement of items by sex, except for lantern, which was endorsed by more men than women. Six of the binary items (cement flooring, television, *boda boda*, refrigerator, bicycle, and iron) had moderate to very good agreement, with Cohen’s κ ranging from 0.68 to 0.85. Four of the binary items (lantern, car, type of toilet facility, and cement walls) had weak agreement with Cohen’s κ ranging from 0.55 to 0.59, while radio (κ = 0.35) and stove (κ = 0.33) had poor agreement. Low κ values for these items were driven by very high and very low prevalence, respectively, despite high percent agreement for these items. Percent agreement was 95% for size of the rainwater harvesting tank (weighted κ = 0.69) and 76% for number of mobile phones (weighted κ = 0.60). Men reported a greater number of continuous assets than women, on average. Agreement in reporting of livestock numbers ranged from good to excellent (Cronbach’s α = 0.97 for chickens, α = 0.76 for cows, and α = 0.69 for goats). Husbands and wives had very good agreement on number of rooms in the home (Cronbach’s α = 0.83) but very poor agreement for land plots (Cronbach’s α = 0.24), with husbands reporting more land ownership on average.

**Table 2:** Concordance between men and women in reporting of specific assets and wealth quintile

	Husbands (N = 404)	Wives (N = 404)	Percent agreement	Inter-rater agreement (95% CI)
Assets coded as binary variables, N (%):*				
Cement floors	107 (26)	105 (26)	94	0.85 (0.79, 0.91)
Television	60 (15)	56 (14)	94	0.76 (0.67, 0.85)
*Boda boda*	60 (15)	53 (13)	93	0.72 (0.62, 0.82)
Refrigerator	12 (3.0)	10 (2.5)	99	0.72 (0.51, 0.93)
Bicycle	257 (64)	253 (63)	86	0.69 (0.62, 0.76)
Iron	26 (6.4)	28 (6.9)	96	0.68 (0.54, 0.83)
Lantern	288 (71)	259 (64)	82	0.59 (0.51, 0.67)
Car	16 (4.0)	11 (2.7)	97	0.58 (0.35, 0.80)
VIP latrine	10 (2.5)	14 (3.5)	97	0.57 (0.33, 0.81)
Cement walls	74 (18)	77 (19)	86	0.55 (0.45, 0.66)
Radio	348 (86)	336 (83)	83	0.35 (0.23, 0.48)
Stove	13 (3.2)	10 (2.5)	96	0.33 (0.074, 0.58)
**Assets coded as ordinal variables, N (%):†**				
Rainwater harvesting tank (L)			95	0.69 (0.56, 0.82)
None	375 (93)	379 (94)		
≤2000	16 (4.0)	17 (4.2)		
2001-5000	1 (0.3)	4 (1.0)		
>5000	12 (3.0)	4 (1.0)		
Mobile phones in household			76	0.60 (0.53, 0.67)
None	36 (8.9)	42 (10)		
One	119 (29)	117 (29)		
Two or more	249 (62)	245 (61)		
**Assets coded as continuous variables, mean (SD):‡**				
Chickens	9.8 (75)	8.5 (63)	-	0.97
Rooms in home	4.4 (1.7)	4.2 (1.6)	-	0.83
Cows	0.9 (3.0)	0.8 (2.7)	-	0.76
Goats	2.4 (3.8)	2.2 (3.0)	-	0.69
Land plots	14.5 (36)	9.1 (14)	-	0.24
**Overall asset index:**			57	0.65 (0.60, 0.70)
1^st^ quintile (wealthiest)	80 (20)	80 (20)	-	
2^nd^ quintile	82 (20)	81 (20)	-	
3^rd^ quintile	80 (20)	81 (20)	-	
4^th^ quintile	81 (20)	82 (20)	-	
5^th^ quintile (poorest)	81 (20)	80 (20)	-	

CI – confidence interval, SD – standard deviation

*Inter-rater agreement for binary items was determined using Cohen’s κ.

†Inter-rater agreement for ordinal assets was determined using weighted κ.

‡Inter-rater agreement for continuous assets was determined using the intraclass correlation coefficient (3,k) (Cronbach’s α). The 95% confidence interval is not relevant for these estimates.

### Agreement for asset index and wealth quintile

**Figure 1:**
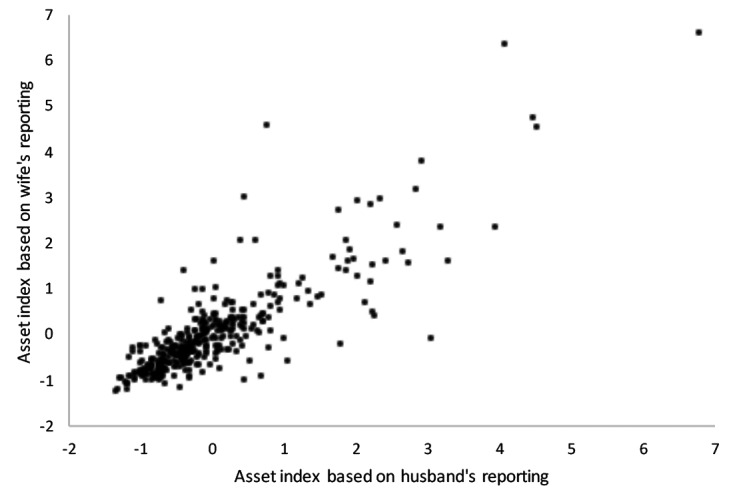
Asset index score based on husband’s reporting vs asset index score based on wife’s reporting.

A scatterplot of household asset wealth index scores based on each wife’s reporting vs the asset wealth index score based on her husbands’ reporting showed a roughly linear correspondence (**Figure 1**). Measurement pairs largely centered around the 45-degree angle line, suggesting that neither men nor women were systematically more likely to over-report or under-report household asset wealth as a whole. Consistent with the scatterplot, the correlation between husbands’ and wives’ asset index scores was positive (Pearson r = 0.85). The figure also indicates greater dispersion at higher values of the asset index scores, suggesting that husbands’ and wives’ asset reporting were more likely to diverge at higher levels of household asset wealth.

Comparing wealth quintiles based on husbands’ reporting to wealth quintiles based on wives’ reporting suggested moderate to good agreement beyond that which would be expected by chance (weighted κ = 0.65). In **Table 3**, cells on the diagonal (boldface type) identify households that were categorized into the same wealth quintile whether asset reporting was derived from husbands vs their wives: in a majority of households (n = 232, 57%), the wealth quintile categorization based on the husband’s asset reporting was equivalent to that based on his wife’s asset reporting. In 361 (89%) households (ie, cells on the diagonal and off-diagonals), the wealth quintile categorization based on the husband’s asset reporting was within one quintile of that based on the wife’s asset reporting.

**Table 3:** Comparison of household wealth quintile classification, based on asset reporting by husbands vs wives (N = 404)

	Wealth index quintile based on asset reporting by husbands, N (row percent)				
**Wealth index quintile based on asset reporting by wives**	**1^st^ (wealthiest)**	**2^nd^**	**3^rd^**	**4^th^**	**5^th^ (poorest)**
1^st^ (wealthiest)	**57 (71)**	14 (17)	6 (7.5)	2 (2.5)	1 (1.3)
2^nd^	13 (16)	**43 (52)**	18 (22)	6 (7.3)	2 (2.4)
3^rd^	9 (11)	19 (24)	**30 (37)**	17 (21)	5 (6.2)
4^th^	0	4 (4.9)	21 (26)	**43 (53)**	13 (16)
5^th^ (poorest)	1 (1.2)	1 (1.2)	6 (7.4)	14 (17)	**59 (73)**

However, in 43 households (11%), asset reporting by husbands vs wives resulted in a difference of two or more wealth quintiles. For example, among the 80 households that would have been categorized as being in the wealthiest quintile based on the wife’s asset reporting, 9 households (11%) would have been categorized as being much poorer (ie, in the 3^rd^, 4^th^, or 5^th^ wealth quintile) had the wealth index scores been based on asset reporting by her husband. Among the 196 households with discrepant wealth quintile categorizations, neither men nor women were more likely to be the respondent reporting greater wealth.

In a multivariable regression model fitted to the data with wealth quintile concordance specified as the dependent variable (equal to 1 if wealth quintile based on the wife’s reporting was the same as wealth quintile based on the husband’s reporting), we identified no statistically significant demographic correlates of concordance (**Table 4**). However, husbands and wives were more likely to provide asset reporting that yielded concordant wealth quintiles at the extremes (ie, the highest and lowest quintiles of asset wealth). In the sensitivity analysis, alternate methods of grouping households by asset index scores other than quintiles yielded slightly higher percent agreement, but the same weighted κ scores: when households were grouped by bottom 40% vs. middle 20% vs. top 40%, percent agreement was 71% with a weighted kappa of 0.65; when households were grouped by bottom 20% vs. middle 60% vs. top 20%, percent agreement was 78% with a weighted kappa of 0.65; and when households were grouped according to tertiles, percent agreement was 68% with a weighted kappa of 0.62.

**Table 4:** Correlates of concordance in wealth quintile (N = 404)

	RR	95% CI
**Wealth quintile based on wife’s reporting:**		
1^st^ (wealthiest)	1.9	1.4, 2.6
2^nd^	1.4	1.0, 2.0
3^rd^	Ref	Ref
4^th^	1.4	1.0, 2.0
5^th^ (poorest)	1.9	1.4, 2.7
**Joint education:**		
Both completed primary	Ref	Ref
Only husband completed primary	0.9	0.7, 1.1
Only wife completed primary	0.9	0.7, 1.2
Neither completed primary	1.1	0.9, 1.3
**Joint age (years):**		
Both <30	1.1	0.8, 1.4
Both 30-49	Ref	Ref
Both >49	1.1	0.9, 1.4
Mixed ages	1.0	0.8, 1.2
**Age gap between husband and wife (5-year increments)**	1.0	1.0, 1.1

CI – confidence interval

*The odds ratios for each of these categorical variables was obtained from a separate log-binomial regression model with a single covariate (joint age, joint education, or joint marital status) fitted to the data. Thus, the estimates in this table were obtained from four log-binomial regression models.

## DISCUSSION

In this population-based study of multi-adult households in rural southwestern Uganda, we found moderate to very good agreement between husbands’ and wives’ reporting of individual household assets. When we categorized households into asset wealth quintiles based on husbands’ vs wives’ reporting of individual household assets, we found that more than half (57%) of households were categorized into the same wealth quintile regardless of the person reporting. However, for a non-trivial proportion of households (43%), their assigned wealth quintile would have differed had the categorization been based on data from husbands’ vs their wives’ reporting. In 11% of households, the difference would have been substantial (ie, two or more quintiles). A kappa statistic of 0.65, which accounts for potential agreement by chance, indicated moderate agreement between husbands’ and wives’ wealth quintile categorization.

Our findings have important implications for the measurement of livelihood status and economic well-being, especially in settings in which asset indices are frequently used – primarily resource-limited settings in which the local economy is dominated by agriculture and animal husbandry. Namely, asking a single household member to report on household assets can lead to significant errors in wealth quintile assignment. In more than one in 10 households, wealth categorization could have differed by two or more quintiles depending on respondent choice: for example, households that would have been categorized as being in the wealthiest quintile based on the wife’s reporting of assets would have been categorized as being in one of the poorest three quintiles based on the husband’s reporting of assets, and vice versa. We could not determine the reasons for discordant reporting, but possible reasons may be related to sex differentials in the incidence of various forms of taxation [29]. Differences, particularly in reporting about land ownership, may also be due to differential knowledge and/or use of assets by men and women in the same household [30-32]. Differences in land plot reporting could also be related to the amount of time respondents spend in particular locations.

Our findings may be generalizable to other resource-limited settings in which asset indices are frequently used to measure wealth. However, some discrepancies, such as differences in land ownership reporting described above, may have specific cultural explanations. Thus, similar studies in other contexts would be informative. Future research should also assess the extent to which measurement and recall exhibit gendered patterning according to the sex of the interviewer vs the study participant’s sex. In a previous study, survey administration by younger women yielded upwardly biased estimates of household asset wealth provided by husbands vs wives [33]. Finally, understanding whether members of polygamous households show distinctive agreement is an area for further inquiry, as our sample did not contain a sufficient number of polygamous households to allow for such an analysis.

There are three main limitations of this study. First, we lacked asset data for some participants, which resulted in excluding 59 of 463 eligible households (15%). Dyads excluded due to missing data were comprised of younger and less educated men and women, and were more likely to be of “mixed ages.” Second, we utilized aggregated predictors to analyze correlates of wealth quintile concordance. These are subject to measurement error and may have masked interesting predictors of concordance.

Third, we were only able to establish the presence of *comparative* differences in reporting by married members of the same household. The study design did not include independent validation of household asset ownership by a third party. For example, in the Give Directly cash transfer experiment, eligibility was contingent on having a home with a thatched roof that was observable by study staff [34]. In practice, research assistants for this study often asked participants to clarify answers that ran contrary to casual observation. Such a situation might arise, for example, if a study participant claimed to have no rainwater harvesting tank when the research assistant could observe a rainwater harvest tank next to the participant’s home. However, research assistants were not specifically instructed to clarify responses, nor did our study design entail having research assistants directly verify the presence or absence of individual assets, or scrutinize any documentation related to ownership. Implementing such procedures would have been possible for some assets but prohibitively expensive for others. For example, in this study setting, it is not uncommon for property owners to own land or keep cattle a significant distance from the primary residence, leaving research assistants unable to verify ownership by direct observation. This limitation is similar to other types of studies with multiple informants but no criterion standard, eg, studies of domestic violence in which both partners are asked about perpetration and victimization [35,36]. An alternative strategy for establishing a baseline level of spousal agreement might be to administer “control” questions, such as the spouse’s age or the number of children in the household, as was done in a novel study by Kolcic et al. [33].

## CONCLUSION

Our study provides a significant contribution to the literature in eliciting household asset wealth information from multiple informants within the same households, thereby establishing that wealth quintile classifications can be sensitive to the choice of informant. Sensitivity analyses established that the degree of agreement was not contingent upon arbitrary decisions about category groupings (eg, quintiles vs tertiles). For investigators who face budgetary constraints in conducting studies in similar settings, and who seek to measure economic status as an important but ancillary variable, the degree of potential misclassification identified in our analysis may be considered acceptable (ie, 89% of households would have been assigned to the same category ± one quintile in either direction). Depending on the aims of their research, investigators may conclude that reasonable estimates of household wealth can be elicited by collecting household asset information from only one adult per household. However, for studies in which accurate measurement of household asset wealth is central to the primary aims of the study, collecting household asset information from multiple informants may be advised.
